# Expression of Concern: Activation of protein phosphatase 2A tumor suppressor as potential treatment of pancreatic cancer

**DOI:** 10.1002/1878-0261.70087

**Published:** 2025-07-08

**Expression of Concern:** Chien W, Sun Q‐Y, Lee KL, Ding L‐W, Wuensche P, Torres‐Fernandez LA, Tan SZ, Tokatly I, Zaiden N, Poellinger L, Mori S, Yang H, Tyner JW, Koeffler HP, Activation of protein phosphatase 2A tumor suppressor as potential treatment of pancreatic cancer. *Mol Oncol*. 2015; 9(4):889–05. https://doi.org/10.1016/j.molonc.2015.01.002.

This Expression of Concern is for the above article, published online January 15, 2015, in Wiley Online Library (wileyonlinelibrary.com), and has been issued by agreement between the journal Editor‐in‐Chief, Kevin Ryan; the Federation of European Biochemical Societies; and John Wiley and Sons Ltd. The Expression of Concern has been agreed upon due to duplications observed between some of the pS9‐GSK3β bands and GAPDH bands presented in Fig. 4E. Although the authors collaborated with the investigation, they were unable to sufficiently address the concerns or produce the raw data for this western blot. Therefore, the journal has decided to issue an Expression of Concern to alert the readers.

